# Luffa Rootstocks Affect Fruit Quality of Grafted White-Fruited Bitter Melon

**DOI:** 10.3390/plants15203192

**Published:** 2026-10-18

**Authors:** Pingping Fang, Haoxin Mu, Haoran Ma, Yang Xiao, Jing Wei, Ting Zhang, Xueyan Wang, Ting Sun, Qinwei Guo

**Affiliations:** 1 Quzhou Key Laboratory for Germplasm Innovation and Utilization of Crop, Quzhou Academy of Agricultural and Forestry Sciences, Quzhou 324000, China; 2 State Key Laboratory of Crop Genetics & Germplasm Enhancement and Utilization, College of Horticulture, Nanjing Agricultural University, Nanjing 210095, China; 3 Jiangsu Academy of Agricultural Sciences Wuxi Branch, Shicheng Road 388, Wuxi 214174, China; sunting@cjlu.edu.cn

**Keywords:** bitter melon, fruit quality, luffa rootstocks, cucurbits grafting

## Abstract

White-fruited bitter melon is a rare species of *Momordica charantia* L., valued for its pearl-like white pericarp. However, limited research is available regarding its supporting cultivation techniques and fruit quality. Two-season field trials were performed using ‘*Kuzhongle*’ as scion to evaluate the applicability of luffa rootstocks and seasonal variation in rootstock-mediated effects. Three luffa inbred lines (SS07, SS11 and SS26) were screened in spring, and the superior SS26 was further compared with the commercial rootstock ‘*Yinzhen No.1*’ in autumn. All rootstocks increased stem diameters, shortened internodes and promoted female flower formation in spring, but their effects on fruit quality varied among genotypes. SS26 exhibited the highest survival rate and significantly enhanced soluble solid content, water content, crude protein content and vitamin C content, while reducing crude fiber content and titratable acidity content. Seasonal comparison revealed consistent quality-modulating effects of SS26 across seasons, except for crude fiber, with a comprehensive performance comparable to the commercial rootstock. Overall, luffa rootstocks consistently modulate vegetative and flowering traits within a season, whereas their impacts on fruit quality show genotypic differences. SS26 exhibits stable and superior grafting performance, providing important material support for bitter melon rootstock breeding research.

## 1. Introduction

The bitter melon (*Momordica charantia* L.) is a high-value, widely consumed cucurbit vegetable with rich health-promoting functions [1]. It serves dual roles as an edible vegetable and medicinal functional crop, generating stable income for producers [2]. It can be consumed as fresh fruit or cooked in various ways, among which stir-frying, steaming and boiling are the three most common cooking methods. This vegetable is valued for its superior eating and sensory qualities, exhibiting crisp flesh texture, high water content, and bitterness. Nutritionally, it is rich in vitamin C, soluble dietary fiber, potassium and multiple bioactive compounds such as saponins and glycosylated terpenoids, which confer outstanding health-promoting properties [3,4]. Growing market demand for its fresh fruit, health-related extracts and processed products continuously promotes its commercial cultivation. White-fruited bitter melon, a distinctive kind of cultivar, renowned for its nearly white skin resembling white pearls [5], was found to contain significantly lower saponin concentrations (0.25%) compared to green cultivars [6]. In recent years, with the breeding and popularization of new hybrid white-fruited bitter melon varieties, including ‘*Quku No.1*’, ‘*Bai yu*’ and ‘*Kuzhongle*’, the cultivation scale of white-fruited bitter melon has expanded gradually [5,7]. However, the rapid expansion of planting scale is not matched with high-efficiency supporting cultivation techniques.

Grafting, as an important asexual plant propagation technology, has been extensively applied in commercial horticulture, particularly for solanaceous and cucurbitaceous vegetables [8,9,10]. This technique involves the combination of two plant components: a scion (above-ground shoot tissue) and a rootstock (underground root tissue), which fuse to form a complete new plant [11]. Benefiting from the rootstock-derived robust roots and excellent growth vigor of the scion, grafted seedlings exhibit vigorous vegetative growth [12]. This grafting practice effectively enhances crop tolerance to biotic and abiotic stresses, boosts fruit yield, optimizes comprehensive fruit quality, as well as regulates flowering and harvest periods [13,14,15]. Grafting has been extensively adopted in commercial bitter melon production, yet scientific information on grafting in bitter melon is meager [16,17].

In commercial bitter melon production, pumpkin (*Cucurbita moschata*) and luffa (*Luffa cylindrica*) seedlings serve as the predominant rootstocks for grafting cultivation [16,17,18,19]. Previous studies have verified the favorable applicability of luffa rootstocks in bitter melon grafting. For instance, grafting bitter melon onto resistant luffa rootstocks can effectively improve plant resistance to Fusarium wilt without causing obvious adverse impacts on fruit quality [20]. Another study demonstrated that grafting bitter melon onto luffa rootstock significantly enhanced the heat tolerance of scion plants. Compared with un-grafted and self-rooted bitter melon, luffa-grafted plants exhibit elevated antioxidant capacity and higher activities of key enzymes related to carbon and nitrogen metabolism [16]. In a comprehensive rootstock screening trial, Chen et al. [17] evaluated the effects of three luffa and four pumpkin rootstocks on the yield, fruit quality, and disease resistance of ‘Lanshan’ bitter melon, and ultimately screened one optimal pumpkin rootstock and two superior luffa rootstocks suitable for this cultivar. Nevertheless, current research on bitter melon grafting predominantly concentrates on improving plant stress resistance and disease tolerance. Systematic investigations regarding the regulatory effects of luffa rootstocks on the nutritional and commercial quality of grafted bitter melon remain insufficient.

Given the lack of specialized luffa rootstock techniques adaptable to white-fruited bitter melon across different growing seasons, this study adopted a two-phase field experiment. We hypothesized that luffa rootstock grafting would improve the fruit quality of white-fruited bitter melon and that such rootstock-mediated effects would differ among luffa genotypes and vary between growing seasons. Three luffa rootstocks were initially evaluated in spring, and the best-performing rootstock screened was further compared with a commercially available luffa rootstock in autumn. The study aimed to quantify grafting effects on major fruit quality traits and provide a theoretical basis and practical guidance for the sustainable high-quality production of white-fruited bitter melon.

## 2. Results

### 2.1. The Influences of Luffa Rootstocks on Survival and Growth of Grafted ‘Kuzhongle’ in Spring

As shown in Table 1, the graft survival rates of white-fruited bitter melon cv. ‘Kuzhongle’ grafted onto the three different luffa rootstocks all exceeded 95.00%, with no significant differences among rootstock treatments. The transplant field survival rates followed a descending order: SS26 (95.00%) > SS07 (90.00%) > SS11 (86.67%). Except for SS26, the other two grafted treatments showed significantly lower transplant survival rates than un-grafted plants. Compared with the un-grafted control, all three graft combinations significantly increased scion stem diameter and reduced main vine internode length. Notably, although grafting onto SS26 significantly shortened the main vine internode length, plant height measured at 7, 14 and 21 days after transplanting remained comparable to that of the un-grafted control (Figure S1). These results suggest that rootstock SS26 facilitates robust vegetative growth of bitter melon.

### 2.2. The Influences of Luffa Rootstocks on Flowering Traits and External Fruit Quality of Grafted ‘Kuzhongle’ in Spring

**Figure 1:**
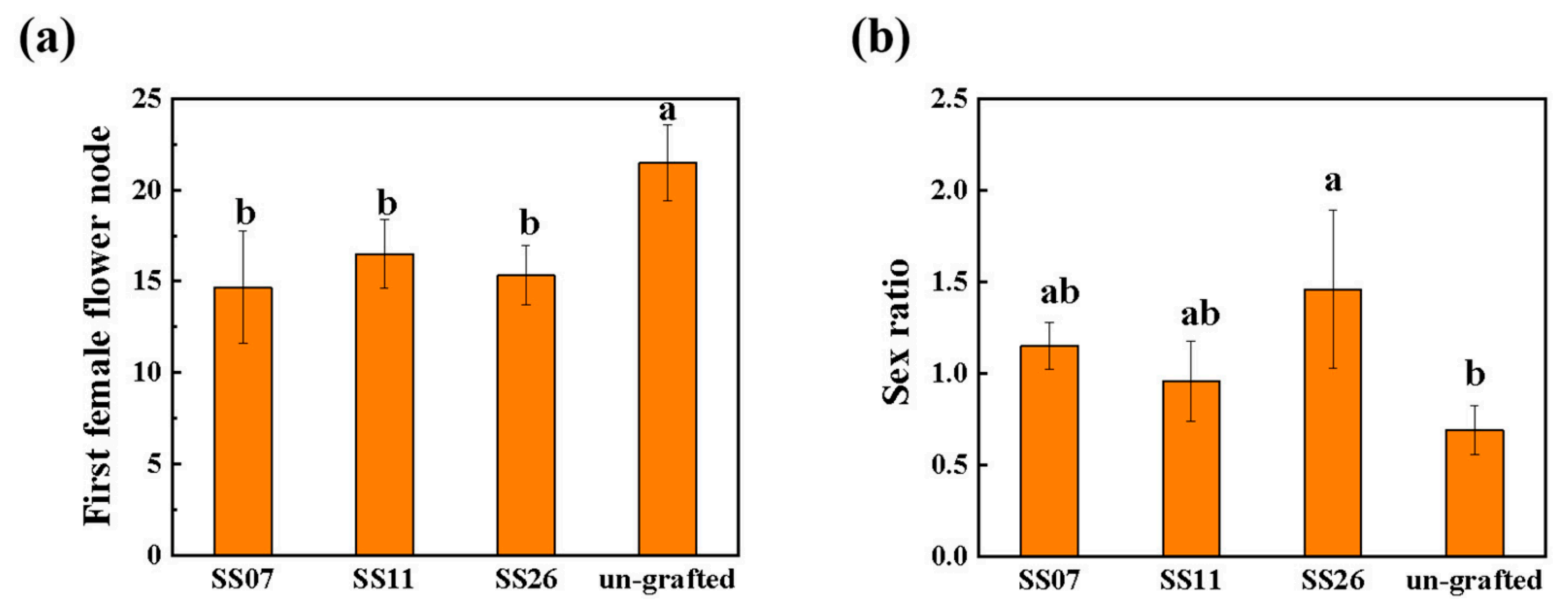
The effects of luffa rootstocks on flower-related traits of grafted ‘*Kuzhongle*’ in spring. (**a**) First female flower node. (**b**) Sex ratio. Values represent the means of three biological replicates ± SD. The average value of six plants within each replicate was used as a single data point for statistical analysis. Different letters indicate significant differences (*p* < 0.05) according to Tukey’s test.

Furthermore, two flowering-related traits, including the first female flower node and sex ratio, were evaluated among treatments. The first female flower node of bitter melon cv. ‘Kuzhongle’ did not differ significantly among luffa rootstock treatments; however, all grafted plants exhibited significantly lower first female flower nodes than the un-grafted control (Figure 1a). The sex ratio of un-grafted plants was 0.72, whereas grafting with rootstocks SS26, SS11 and SS07 elevated this value to 1.45, 0.95 and 1.15, respectively (Figure 1b). Since white pericarp is a distinctive fruit characteristic of white-fruited bitter melon and an important trait influencing consumer preference, we further investigated whether grafting with luffa rootstocks affects fruit skin color. Our results showed that the color parameters L*, a* and b* exhibited no significant differences among ‘*Kuzhongle*’ plants grafted onto different rootstocks and the non-grafted control (Table 2). Similarly, no significant differences were observed in fruit length, fruit diameter, single fruit weight, or pedicel length across all treatments (Table 2).

### 2.3. The Influences of Luffa Rootstocks on Internal Fruit Quality of Grafted ‘Kuzhongle’ in Spring

**Figure 2:**
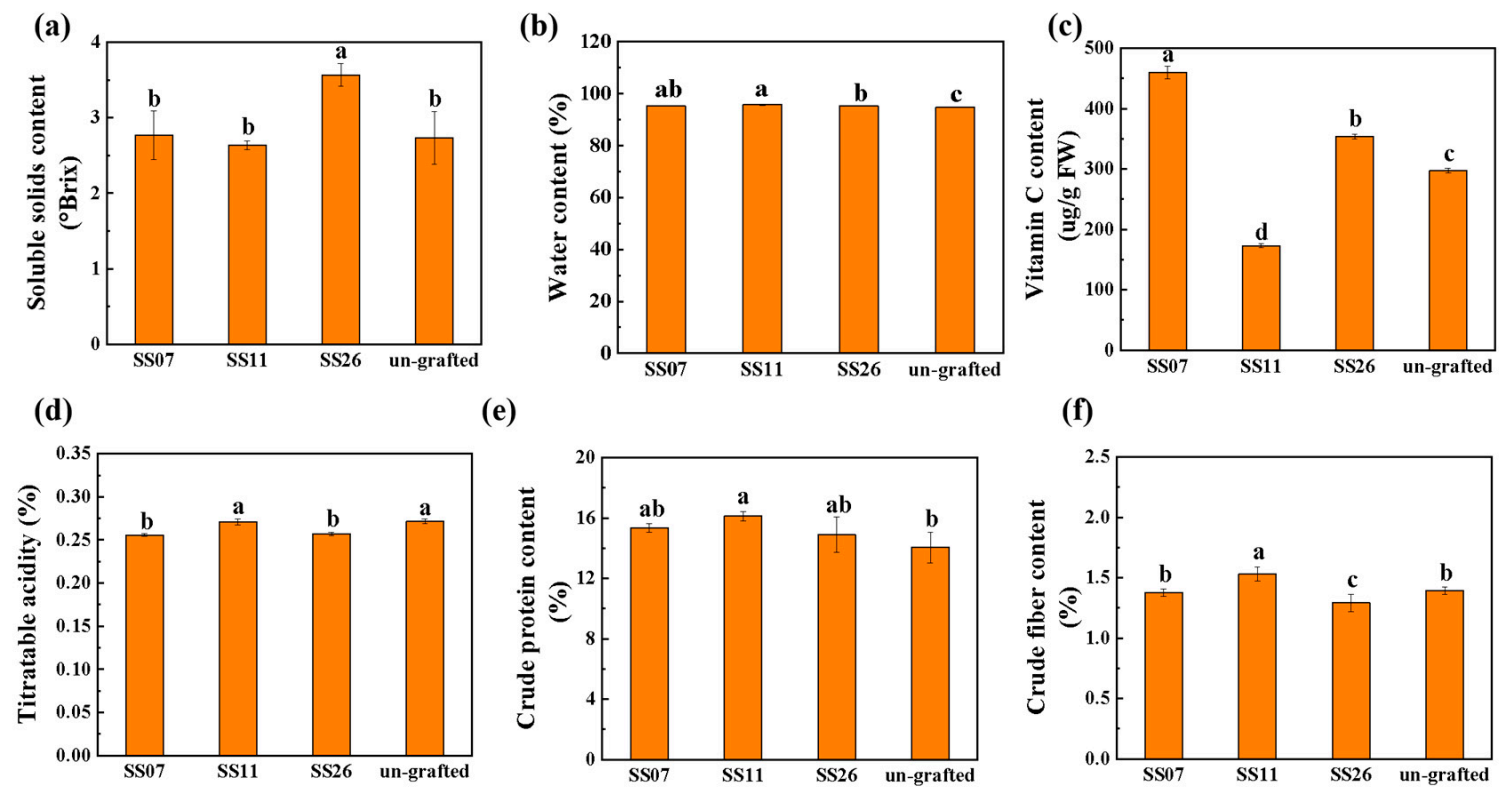
The effects of luffa rootstocks on internal fruit quality of grafted ‘*Kuzhongle*’ in spring. (**a**) Soluble solids content; (**b**) water content; (**c**) vitamin C content; (**d**) titratable acidity; (**e**) crude protein content; (**f**) crude fiber content. Values represent the means of three biological replicates ± SD. Six fruits were pooled as one replicate sample. Different letters indicate significant differences (*p* < 0.05) according to Tukey’s test.

Grafting with different luffa rootstocks exerted significant effects on all measured indicators of internal fruit quality in white-fruited bitter melon (Figure 2). The content of soluble solids in fruits grafted onto rootstock SS26 was significantly higher than that in fruits from all other treatments (Figure 2a). The lowest water content was detected in the un-grafted control, which was significantly lower than that of the three grafted treatments (Figure 2b). The highest vitamin C content was determined in fruits from plants grafted onto SS07 (459.7 μg/g), followed by those grafted onto SS26 (354.2 μg/g). However, fruits from SS11-grafted plants had the lowest vitamin C content (172.6 μg/g), which was lower than that of the un-grafted plants (Figure 2c). The titratable acidity was expressed as equivalent malic acid. As shown in Figure 2d, the titratable acidity of fruits from SS11-grafted and non-grafted plants was significantly higher than that of SS07- and SS26-grafted plants. The highest crude protein content was observed in SS11-grafted fruits, whereas the un-grafted control exhibited the lowest value (Figure 2e). Regarding crude fiber content, SS11-grafted fruits showed the highest level, while SS26-grafted fruits had the lowest (Figure 2f). Collectively, these results indicate that luffa rootstocks exerted differential regulatory effects on internal fruit quality traits, including soluble solids, vitamin C, acid, crude protein and crude fiber contents, in white-fruited bitter melon. Among the tested rootstocks, SS26 shows potential for improving fruit quality for fresh consumption by increasing soluble solids, water content and vitamin C content while reducing titratable acidity and crude fiber accumulation.

### 2.4. The Influences of SS26 Rootstock on Survival and Growth of Grafted ‘Kuzhongle’ in Autumn

Since SS26 showed the highest transplant survival rate and improved fresh fruit quality in spring cultivation, a further grafting experiment was conducted to determine the stability of its effects. Meanwhile, a commercial rootstock ‘*Yinzhen No.1*’ (YZ) was included as a reference for comparison. As shown in Table 3, white-fruited bitter melon cv. ‘Kuzhongle’ grafted onto YZ achieved 100% for both graft survival and transplant survival rates, which were 2.67% and 8.33% higher than those grafted onto SS26, respectively. Consistent with the results obtained from spring, grafting with SS26 significantly reduced main vine internode length. However, unlike the spring experiment, grafting with SS26 did not significantly affect stem diameter under the current experimental conditions. Similarly, the commercial rootstock YZ reduced main vine internode length but had no significant effect on stem diameter compared with un-grafted plants.

### 2.5. The Influences of SS26 Rootstock on Fruit Quality of Grafted ‘Kuzhongle’ in Autumn

**Figure 3:**
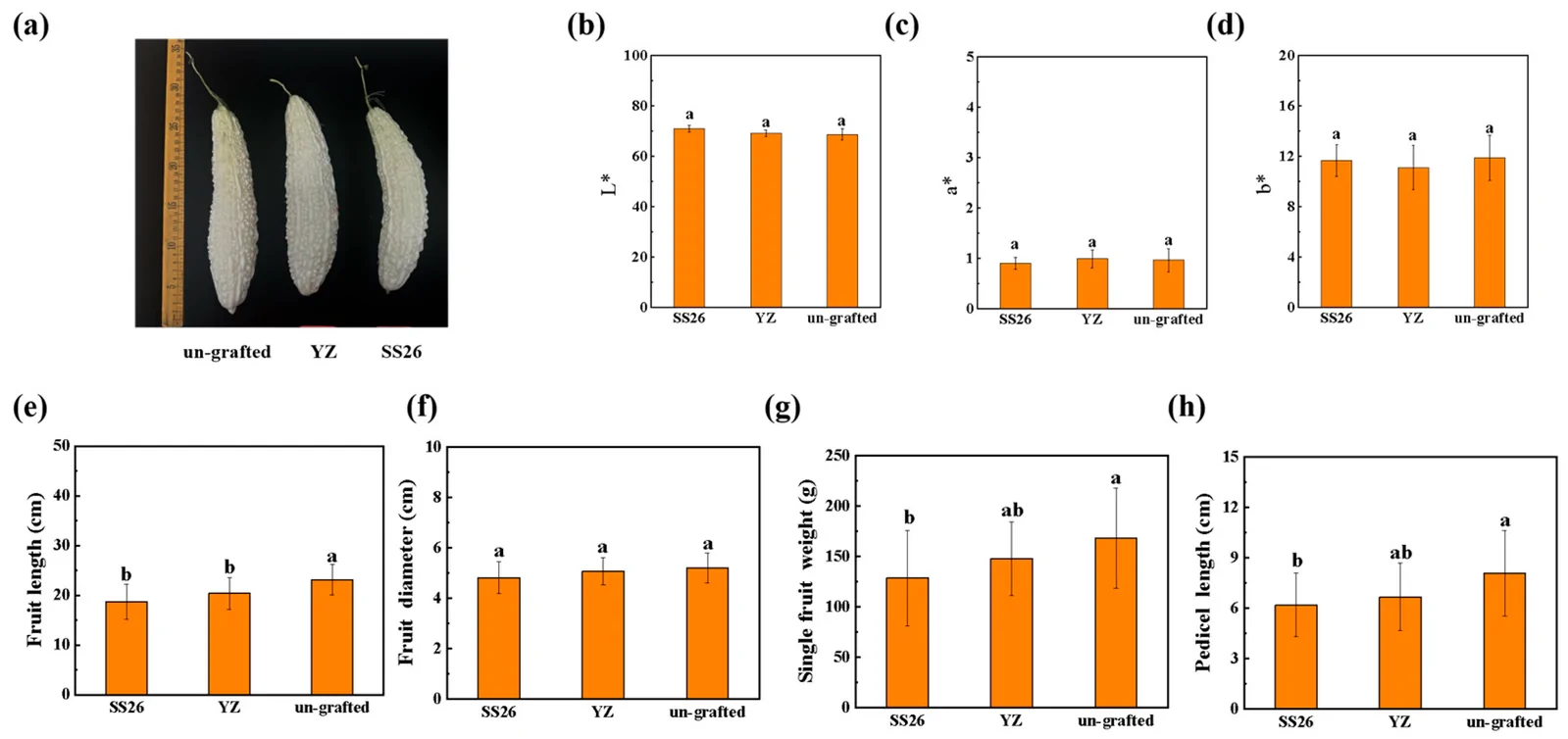
The effects of luffa rootstocks on external fruit quality of grafted ‘*Kuzhongle*’ in autumn. (**a**) Photos of harvest fruits; (**b**–**d**) L* (**b**), a* (**c**) and b* (**d**) detected using a color meter; (**e**) fruit length; (**f**) fruit diameter; (**g**) single fruit weight; (**h**) pedicel length. Values represent the means of three biological replicates ± SD. The average value of six fruits within each replicate was used as a single data point for statistical analysis. Different letters indicate significant differences (*p* < 0.05) according to Tukey’s test.

Different from the results obtained from the spring experiment, grafting using luffa stocks affected several fruit external traits, including fruit length, single fruit weight and pedicel length (Figure 3). Un-grafted plants produced significantly longer fruits than those grafted onto SS26 and YZ rootstocks, whereas fruit diameter showed no significant differences among all treatments. The maximum single fruit weight and pedicel length were recorded in un-grafted bitter melon plants, which were significantly greater than those of SS26-grafted plants. The YZ-grafted group displayed intermediate values for both indicators.

**Figure 4:**
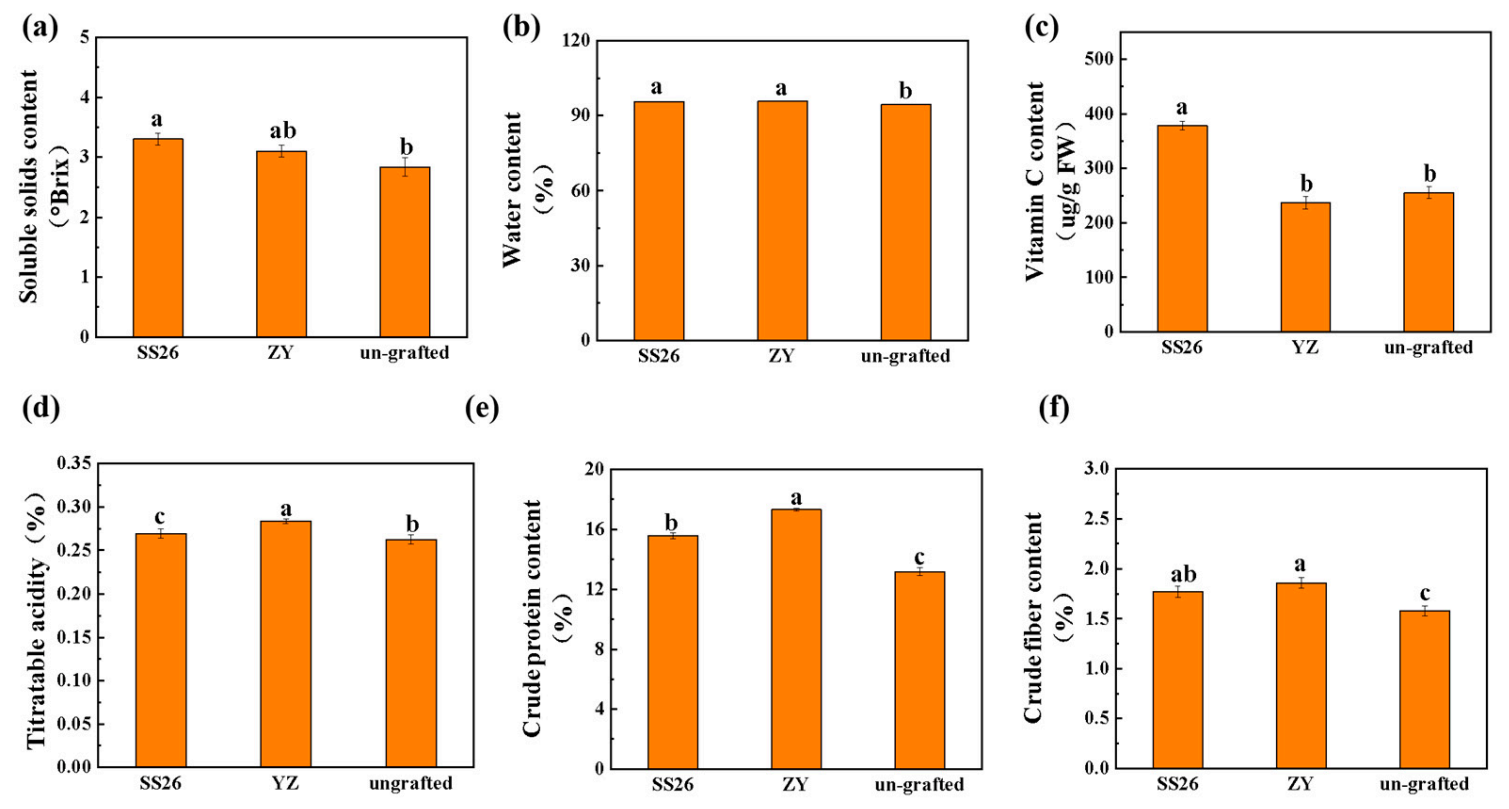
The effects of luffa rootstocks on internal fruit quality of grafted ‘*Kuzhongle*’ in autumn. (**a**) Soluble solids content; (**b**) water content; (**c**) vitamin C content; (**d**) titratable acidity; (**e**) crude protein content; (**f**) crude fiber content. Values represent the means of three biological replicates ± SD. Six fruits were pooled as one replicate sample. Different letters indicate significant differences (*p* < 0.05) according to Tukey’s test.

In autumn, SS26 and YZ exhibited similar effects on most internal fruit quality traits, including soluble solids content, water content, crude protein content and crude fiber content. The highest soluble solids content and vitamin C content were recorded in SS26-grafted plants, both of which were significantly higher than those in the un-grafted control (Figure 4a). In addition, vitamin C content in SS26-grafted plants was significantly higher than that in YZ-grafted plants (Figure 4c). The water content of un-grafted fruits was significantly lower than that of fruits from both SS26- and YZ-grafted plants (Figure 4b). The titratable acidity was significantly reduced by SS26 grafting but increased by YZ grafting compared with the un-grafted control (Figure 4d). Both crude protein and crude fiber contents were highest in YZ-grafted plants, followed by SS26-grafted plants, whereas the lowest values were detected in the un-grafted plants (Figure 4e,f). These results indicate that the fruit quality-modifying effects of SS26 were largely consistent between spring and autumn cultivation, and its regulatory performance was comparable to that of the commercial rootstock YZ in terms of most fruit quality traits.

## 3. Discussion

Grafting is an extensively adopted technique in cucurbit production to enhance plant growth, stress adaptation and fruit yield [10,13,20,21,22]. Nevertheless, grafting commonly leads to a decline in fruit quality [23]. Previous studies have confirmed that luffa germplasm possesses excellent graft compatibility with bitter melon and serves as a dominant rootstock in bitter melon cultivation, attributed to its strong resistance to Fusarium wilt and robust tolerance to heat and waterlogging [22,24]. However, available information regarding the quality-modulating effects of luffa rootstocks on bitter melon, especially white-fruited cultivars, remains limited. In this study, three luffa rootstock genotypes (SS07, SS11 and SS26) were used to graft white-fruited bitter melon cv. ’*Kuzhongle*’ to evaluate their differences in grafting compatibility, reproductive growth performance and fruit quality regulation. Furthermore, the elite rootstock SS26 was assessed for seasonal adaptability and compared with the commercial rootstock YZ to clarify its practical application potential and targeted production advantages in white-fruited bitter melon production.

The effectiveness of grafting largely depends on the compatibility between scion and rootstock genotypes [10,25]. In the present study, all tested luffa rootstocks exhibited high graft compatibility with white-fruited bitter melon cv. ‘*Kuzhongle*’, with graft survival rates exceeding 95% under spring cultivation. Among them, SS26 achieved the highest graft and transplant survival rates, reflecting superior graft affinity compared with SS07 and SS11. The enhanced survival performance of SS26 may be associated with better vascular reconnection, efficient water transport and stronger root activity after graft union formation, which have been recognized as critical factors determining graft success in cucurbit crops [26,27]. Beyond graft survival, rootstock genotype markedly influenced vegetative architecture, flowering and fruit quality. Relative to the un-grafted controls, all luffa rootstocks increased stem diameters and shortened internodes on the main vine, while lowering the node of the first female flower and increasing female flower proportion. Notably, SS26, which improved early plant establishment and maintained normal plant height despite shortened internodes, generated the highest sex ratio. Such regulation may be attributed to root-derived nutrient acquisition and long-distance hormonal signals transported from the rootstock as detected in other grafted vegetables [28,29]. An elevated sex ratio represents a critical agronomic advantage for bitter melon production, as it directly determines potential fruit-setting capacity, though actual yield improvements require further field validation.

Fruit quality is a major consideration for the commercial production of grafted vegetables because rootstocks can either improve or negatively affect scion fruit characteristics [23,30]. In this study, grafting with luffa rootstocks did not alter the white pericarp color or major external fruit traits during spring cultivation. However, large genotypic differences existed for internal quality attributes. SS26 increased soluble solids, water content and vitamin C while decreasing titratable acidity and crude fiber, jointly improving fruit tenderness, juiciness and nutritional value. The distinct effects among SS07, SS11 and SS26 further indicate that rootstock selection is a key factor determining fruit quality outcomes after grafting. Direct comparison with the commercial rootstock YZ further illuminated the practical positioning of SS26. YZ exhibited marginally higher graft survival rates, confirming its reliable commercial graft compatibility. However, SS26 delivered comparable or superior performance for key fresh-market quality traits. SS26 produced higher soluble solids and vitamin C, whereas YZ favored accumulation of crude protein and crude fiber. The differences between SS26 and YZ may reflect genetic variation in root system characteristics and rootstock-mediated metabolic regulation.

The rootstock-mediated effects exhibited by SS26 are subject to seasonal environmental fluctuations, highlighting genotype-by-environment interactions. SS26 increased stem diameter under spring conditions, whereas this vegetative advantage was not observed in autumn. Similarly, grafting showed limited effects on external fruit traits in spring but significantly influenced fruit length, single fruit weight, and pedicel length in autumn. These differences indicate that the interaction between rootstock genotype and seasonal environmental factors may determine the final phenotype of grafted plants. Seasonal variation in temperature, solar radiation and humidity reshapes root activity, mineral uptake, source–sink balance and carbohydrate partitioning, thereby reshaping grafted-plant phenotypes. Although SS26 consistently improved fresh-consumption-related fruit traits across both growing seasons, the extent of changes in external fruit quality varied between seasons. This suggests that rootstock-mediated fruit quality is controlled by both genetic and environmental factors rather than being solely determined by genotype. Previous studies have demonstrated that grafting can regulate fruit quality through modifications of mineral absorption, photosynthetic efficiency, water transport and hormone signaling, all of which are highly responsive to environmental conditions [29,31]. Therefore, multi-season and multi-location evaluations are necessary before the large-scale application of specific rootstocks.

This study provides convincing phenotypic evidence that SS26 is a promising luffa rootstock for high-quality production of white-fruited bitter melon; however, several limitations remain. First, yield-related traits were not quantified. Since yield improvement is one of the primary objectives of vegetable grafting, future studies should determine whether the increased female flower ratio and enhanced vegetative growth induced by SS26 can ultimately translate into yield advantages. Second, broader screening of luffa germplasm resources and breeding populations may facilitate the identification of rootstocks with stronger graft compatibility and quality-enhancing capacity. In addition, comparisons with widely used pumpkin rootstocks are needed to determine whether luffa rootstocks possess unique advantages for bitter melon production. Finally, the physiological and molecular mechanisms underlying SS26-mediated improvements in fruit quality remain unclear. Integrating physiological analyses with transcriptomic, metabolomic and root system investigations will provide deeper insights into how luffa rootstocks regulate scion growth and fruit quality.

## 4. Materials and Methods

### 4.1. Plant Material and Grafting

In this study, four luffa rootstocks, including SS07, SS11, SS26 and ‘*Yinzhen No. 1*’(YZ), and one scion cultivar, ‘*Kuzhongle*’, were used. SS07, SS11 and SS26 are local landraces originally collected from Zhejiang province and purified into advanced luffa inbred lines. Their seeds were produced and saved at the Quzhou Academy of Agricultural and Forestry Sciences. The detailed information of these luffa lines is summarized in Table S1. The white-fruited bitter melon ‘*Kuzhongle*’ and the commercial rootstock ‘*Yinzhen No.1*’ were purchased from Letuzhongye company (Quzhou, China). YZ was bred by Fujian Academy of Agricultural Sciences. It exhibits high graft compatibility with various bitter melon cultivars, with a graft survival rate greater than 90% and a 100% transplant survival rate [32]. This commercial variety also has high tolerance to Fusarium wilt and has been used for bitter melon production in Zhejiang, China, for several years.

The experiments were conducted at the Donghu Science and Technology Innovation Base of the Quzhou Academy of Agricultural Sciences, Zhejiang Province (118°01′ E, 28°14′ N) in 2025. Seeds were sown in 50-cell plug trays. Before sowing, the seeds were treated by cutting the seed coat opposite the embryo site, followed by soaking in water and incubating at 30 °C for 24 h to achieve uniform germination. The pretreated seeds were directly sown into 50-cell plug trays containing an autoclaved peat moss and perlite soil mixture (3:1, *v*/*v*) and cultivated in a climate chamber under dark conditions at 28 °C. After approximately one-third of the seedlings had emerged, the conditions were adjusted to a 14 h light/10 h dark photoperiod, with temperatures of 28 °C/20 °C (day/night), a light intensity of 20,000 Lux and relative humidity of 60–70%.

When the cotyledons of the scion seedlings had fully opened and the first true leaves of rootstock seedlings had fully unfolded, bitter gourd seedlings were grafted onto luffa rootstocks using the splice grafting method [33]. The grafted plants were maintained at a condition of approximately 95% relative humidity for five days with a day/night temperature cycle of 26 °C/18 °C. The light intensity was maintained at 3000 Lux. Subsequently, the plants were transferred to the climate chamber for further cultivation.

### 4.2. Post-Transplanting Cultivation Conditions

Grafted plants were transplanted on 10 April 2025 and 4 September 2025, in an 8 m-wide standard single-span plastic greenhouse using a double-row, high-ridge system covered with plastic film. The wide and narrow row spacings were 150 cm and 60 cm, respectively. Treatments were arranged in a randomized complete block design with three replicates, and each replicate contained 20 plants. Bitter melon plants were trained using a single-vine system for vertical cultivation in the greenhouse. Only the main vine was retained, and all lateral shoots were regularly removed. The main vine was suspended and tied to trellis lines to support upward growth. Hand pollination was conducted during the flowering period. Two fruits were retained per plant. Fruits were harvested 10 days after pollination, and harvested samples were used for subsequent fruit quality analysis. Conventional drip irrigation, fertilization and pest and disease management were implemented throughout the growing season. Crop management ceased after fruit sample collection. Temperature and relative humidity data are presented in Table S2.

### 4.3. Graft Survival Rate and Transplant Survival Rate

The graft survival rate was assessed 10 days after the grafting. Grafted plants were regarded as survived if the scion leaves exhibited turgidity and obvious expansion. Fifteen days after transplanting, the transplant survival rate was calculated by counting the number of surviving plants. For graft survival rate assessment, 80 and 75 plants per replicate were used in spring and autumn, respectively. For transplant survival rate, 20 plants per replicate were evaluated in each replicate.

Graft survival rate (%) = (survival plants number/total number of grafted plants) × 100%

Transplant survival rate (%) = (survival plants number/total number of transplanted plants) × 100%

### 4.4. Measurements of Growth- and Flower-Related Traits

Stem diameter and internode length of the main vine were measured at 40 d after transplanting. Plant height was measured at 7, 14 and 21 d after transplanting in the spring season. The average length from the 1st to the 10th node on the main vine was taken as the internode length. Stem diameter at 10 cm above ground was measured using a digital vernier caliper (CJW888, AIRAJ, Qingdao, China). The node position of the first female flower borne on the main vine of bitter gourd was recorded as the first female flower node. Female and male flowers were counted on 10 consecutive nodes starting from the first female flower node. Sex ratio was calculated as the number of female flowers divided by the number of male flowers.

### 4.5. External Quality Measurements of Fruits

Fruits were harvested 10 days after pollination. Six fruits were randomly collected from each replicate, with a maximum of one fruit sampled per plant. The external quality factors, including fruit length (cm), diameter (cm), single fruit weight (g), pedicel length (cm) and fruit color, were measured. Fruit color (L*, a* and b*) was detected using a color meter (CS-10, Color Spectrum Technology, Hangzhou, China). The average value of six fruits within each replicate was used as a single data point for statistical analysis.

### 4.6. Measurements of Water and Soluble Solids Content

Water content was detected using the oven-drying gravimetric method according to a published Chinese standard [34]. Soluble solids content was determined by the refractometric method. A droplet of juice from mature fruit was placed over the prism of a digital pocketable refractometer PAL-1 (Atago, Guangzhou, China). Results were expressed as °Brix.

### 4.7. Measurements of Vitamin C Content

Vitamin C content was measured using a kit (Suzhou Keming Biotechnology Co., Ltd., Suzhou, China) based on the fast blue salt B spectrophotometric method [35]. Fresh samples (0.1 g) were homogenized with 1 mL of acetic acid solution (2 mol/L) and then transferred to a 1.5 mL centrifuge tube. Subsequently, the samples were centrifuged at 8000× *g* for 5 min at 4 °C. The resulting supernatant was assayed for vitamin C content at 420 nm according to the protocol provided with the assay kit.

### 4.8. Measurements of Crude Protein Content

Crude protein content was measured according to the Chinese national food-safety standard [36]. Briefly, dried fruit samples were ground and passed through a 40-mesh sieve. Approximately 0.3 g of each sample was digested with concentrated sulfuric acid and hydrogen peroxide using a graphite digestion system with a programmed heating procedure: 100 °C for 10 min, 280 °C for 10 min and 400 °C for 40 min. After cooling, 1 mL of hydrogen peroxide was added for decolorization, followed by a second digestion procedure (200 °C for 10 min, 320 °C for 30 min and 400 °C for 30 min). After cooling again, an additional 0.5 mL of hydrogen peroxide was added, and the samples were further digested (200 °C for 10 min, 320 °C for 30 min and 400 °C for 60 min) until the digestion solution became clear. The digested solutions were analyzed using an automatic Kjeldahl distillation–titration system (K1100, Haineng, Qindao, China).

### 4.9. Measurements of Titratable Acidity

Titratable acidity was determined according to the Chinese national food-safety standard [37] with minor modifications for sample extraction. Briefly, 1 g of fresh sample was homogenized with 10 mL of distilled water, and the homogenate was subsequently centrifuged at 8000× *g* for 10 min at 4 °C. A volume of 0.5 mL supernatant was transferred into a small beaker and diluted with 9.5 mL of distilled water. Subsequently, 10 μL Reagent I (1% phenolphthalein indicator: 1 g phenolphthalein dissolved in a mixture of 60 mL absolute ethanol and 40 mL distilled water) was added. The mixture was titrated with 0.01 mol/L NaOH solution until a persistent faint pink color appeared, and the consumed volume of NaOH solution was recorded. Titratable acidity was calculated and represented the content of equivalent malic acid. V: volume, mL; C: concentration, mol/L; W: fresh weight of sample, g.

Titratable acidity (%) = (V_NaOH_× C_NaOH_) × 10^−3^ × 67 × 100)/W.

### 4.10. Measurements of Crude Fiber Content

Crude fiber content was measured according to the Chinese national standard [38]. Briefly, an appropriate 10 g fresh sample was accurately weighed and recorded as W_1_. The sample was digested using 1.25% H_2_SO_4_ at 150 °C for 40 min. The residue was further digested with 50 mL of 1.25% NaOH at 150 °C for 40 min. The mixture was filtered through a six-layer gauze, and the residue was washed with water until non-alkaline. The residue was washed twice with 10 mL of 95% ethanol and filtered. The obtained residue was dried at 105 °C to a constant weight, which was recorded as W_2_. Subsequently, the crucible containing the dried residue was incinerated in a muffle furnace at 500 °C for 3 h, and the ash weight together with the crucible was recorded as W_3_.

Crude fiber content (%) = (W_2_ − W_3_)/W_1_ × 100%

### 4.11. Data Analysis

The graft and transplant survival rates in autumn were compared using a *t*-test (*p* < 0.05). The other datasets were subjected to one-way ANOVA, followed by Tukey’s test at *p* < 0.05. Prior to ANOVA, residual normality and homogeneity of variance were checked. The treatment group was defined as the fixed factor. Data are shown as the means of three replicates. All statistical analyses were performed using the SPSS package (SPSS 19.0, Chicago, IL, USA).

## 5. Conclusions

This study demonstrated that luffa rootstocks exhibited high graft compatibility with white-fruited bitter melon and significantly affected plant growth, flower-related traits and fruit quality. Among the tested genotypes, SS26 showed superior grafting performance, with higher survival rates, enhanced vegetative growth and an increased sex ratio. Moreover, SS26 consistently improved key fresh-consumption fruit quality traits across seasons. Compared with the commercial rootstock YZ, SS26 showed comparable graft compatibility and greater advantages in quality improvement, highlighting its potential for high-quality, white-fruited bitter melon production. However, seasonal environmental variation influenced the SS26-mediated effects, indicating the importance of genotype-by-environment interactions. Future work will include more scion genotypes and commercial rootstocks to validate our findings, together with further studies on yield performance and underlying physiological mechanisms for wider application.

## Figures and Tables

**Table 1:** Evaluation of scion–rootstock graft compatibility in the spring season.

Rootstock	Graft Survival Rate (%)	Transplant Survival Rate (%)	Stem Diameter (mm)	Internode Length (cm)
SS07	95.00 ± 2.50 a	90.00 ± 0.00 bc	9.26 ± 0.12 ab	30.11 ± 3.98 b
SS11	96.25 ± 3.30 a	86.67 ± 5.77 c	9.03 ± 0.26 b	32.20 ± 7.45 b
SS26	97.50 ± 2.50 a	95.55 ± 5.00 ab	9.41 ± 0.16 a	33.82 ± 4.87 b
un-grafted		100.00 ± 0.00 a	8.54 ± 0.32 c	40.77 ± 2.98 a

Values represent the means of three biological replicates ± SD. For the measurement of stem diameter and internode length, the average value of six plants within each replicate was used as a single data point for statistical analysis. Different letters indicate significant differences (*p* < 0.05) according to Tukey’s test.

**Table 2:** The effects of luffa rootstocks on external fruit quality of grafted ‘*Kuzhongle*’ in spring.

Rootstock	L*	a*	b*	Fruit Length (cm)	Fruit Diameter (cm)	Single Fruit Weight (g)	Pedicel Length (cm)
SS07	72.68 ± 2.09 a	1.16 ± 0.35 a	10.03 ± 3.00 a	23.54 ± 2.98 a	4.37 ± 0.48 a	152.24 ± 11.65 a	8.76 ± 1.65 a
SS11	75.61 ± 1.55 a	1.15 ± 0.20 a	11.86 ± 1.77 a	26.36 ± 1.24 a	4.75 ± 0.38 a	159.27 ± 22.75 a	9.13 ± 2.15 a
SS26	76.84 ± 0.78 a	1.12 ± 0.14 a	10.79 ± 2.41 a	26.23 ± 2.96 a	4.43 ± 0.37 a	159.43 ± 15.55 a	10.21 ± 1.66 a
un-grafted	72.78 ± 0.65 a	1.16 ± 0.22 a	12.31 ± 1.12 a	26.80 ± 2.76 a	4.36 ± 0.38 a	164.56 ± 21.93 a	10.27 ± 1.56 a

Values represent the means of three biological replicates ± SD. The average value of six fruits within each replicate was used as a single data point for statistical analysis. Different letters indicate significant differences (*p* < 0.05) according to Tukey’s test.

**Table 3:** Evaluation of scion–rootstock graft compatibility in the autumn season.

Rootstock	Graft Survival Rate (%)	Transplant Survival Rate (%)	Stem Diameter (mm)	Internode Length (cm)
SS26	97.33 ± 1.33 b	91.67 ± 2.88 b	10.68 ± 0.79 ab	40.89 ± 3.27 b
YZ	100.00 ± 0.00 a	100.00 ± 0.00 a	10.71 ± 0.25 b	39.12 ± 2.37 b
un-grafted			10.62 ± 0.27 a	51.04 ± 4.73 a

Values represent the means of three biological replicates ± SD. For the measurement of stem diameter and internode length, the average value of six plants within each replicate was used as a single data point for statistical analysis. Different letters indicate significant differences (*p* < 0.05) according to Tukey’s test or *t*-test.

## Data Availability

All data generated or analyzed during this study are included in this published article.

## Supplementary Materials

The following supporting information can be downloaded at: https://www.mdpi.com/article/10.3390/plants15203192/s1, Figure S1: The plant height recorded in bitter melon plants grafted onto various luffa stocks in spring; Table S1: Characteristics of luffa inbred lines used in this study; Table S2: Temperature and humidity information during spring and fall season in 2025.
