# Supplementary material for: Luffa Rootstocks Affect Fruit Quality of Grafted White-Fruited Bitter Melon
Source: Plants (Basel). 2026 Oct 18;15(20):3192. doi: 10.3390/plants15203192 (PMC13709001; doi:10.3390/plants15203192)
Supplement: Supplementary file 1 [file plants-15-03192-s001.zip › plants-4560695-supplementary.pdf]

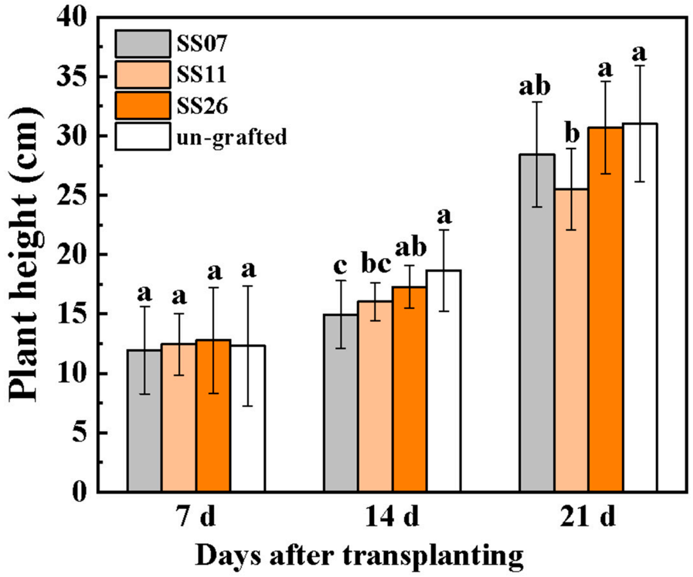

Figure S1. The plant hight recorded in bitter melon plants grafted onto various luffa stocks in spring. Values represent the means of three biological replicates±SD. The average value of six fruits within each replicate was used as a single data point for statistical analysis. Different letters indicate significant differences ($p < 0.05$) according to Tukey's test.

Table S1. Characteristics of luffa inbred lines used in this study

Line name	Internode length (cm)	Stem diameter (mm)	First female flower node	Fruit color	Salt tolerance	Resistance evalution	
						Fusarium wilt	downy mildew
SS07	19.73±2.34	10.51±0.52	14±2.58	Green	Middle	High	Middle
SS11	21.36±0.94	10.03±0.55	12.25±3.1	Green	High	High	High
SS26	17.07±1.56	10.18±0.82	13.87±2.16	Light green	High	High	High

Data were obtained from a preliminary characterization experiment, no statistical multiple comparison was performed. Values are means±SD (n=6). High indicates a previously evaluated salt injury index or disease index of less than 30%, whereas Middle means an index ranging from 30% to 50%.

Table S2 Temperature and humidity information during spring and fall season in 2025

Time	Temperature/℃	Daily relative humidity/%
10/04/2025	17.5~30.3	72.3
11/04/2025	18.5~33.5	69.4
12/04/2025	10.3~23.5	76.4
13/04/2025	5.8~21.5	46.3
14/04/2025	11.2~26.1	45.7
15/04/2025	13.1~27.2	56.2
16/04/2025	14.0~29.9	58.9
17/04/2025	17.2~24.7	75.9
18/04/2025	15.1~31.9	76.1
19/04/2025	20.8~24.5	89.2
20/04/2025	19.1~29.0	76.8
21/04/2025	20.4~29.4	84.8
22/04/2025	19.6~23.3	92.3
23/04/2025	17.9~22.4	83.4
24/04/2025	15.8~19.8	90.9
25/04/2025	14.0~21.4	73.8
26/04/2025	11.3~21.6	61.4
27/04/2025	15.2~21.3	77.9
28/04/2025	16.9~26.3	76.0
29/04/2025	18.2~27.8	73.2
30/04/2025	17.7~25.4	80.2
01/05/2025	17.8~27.6	75.3
02/05/2025	15.9~31.3	69.1
03/05/2025	19.6~29.9	65.0
04/05/2025	17.9~27.7	58.5
05/05/2025	17.4~26.3	86.2
06/05/2025	16.9~29.1	70.9
07/05/2025	17.9~30.3	58.8
08/05/2025	19.5~23.5	89.2
09/05/2025	18.7~26.4	82.8
10/05/2025	16.1~27.4	63.1
11/05/2025	15.8~26.8	76.7
12/05/2025	13.7~28.6	76.9
13/05/2025	16.9~30.6	77.2
14/05/2025	18.3~32.6	77.4
15/05/2025	21.9~29.2	77.6
16/05/2025	21.1~23.7	77.9
17/05/2025	20.7~29.4	78.1
18/05/2025	20.5~23.1	78.4
19/05/2025	19.6~29.0	78.6
20/05/2025	22.2~27.9	78.9

21/05/2025	22.3~29.9	79.1
22/05/2025	22.9~30.8	79.4
23/05/2025	19.6~26.5	79.6
24/05/2025	17.8~24.9	79.9
25/05/2025	19.3~24.0	80.1
26/05/2025	18.9~26.7	80.3
27/05/2025	18.0~28.2	80.6
28/05/2025	17.4~20.5	80.8
29/05/2025	15.8~24.7	81.1
30/05/2025	17.0~28.8	81.3
04/09/2025	28.5~37.9	56.1
05/09/2025	28.3~38.5	56.8
06/09/2025	27.5~39.5	53.5
07/09/2025	28.8~39.1	56.8
08/09/2025	28.7~38.9	54.3
09/09/2025	27.7~37.2	75.3
10/09/2025	25.7~32.4	80.8
11/09/2025	25.3~33.0	77.3
12/09/2025	26.1~35.3	70.6
13/09/2025	27.0~35.2	74.5
14/09/2025	26.0~35.1	75.9
15/09/2025	26.2~36.0	76.5
16/09/2025	26.8~37.2	74.1
17/09/2025	27.3~37.2	70.8
18/09/2025	26.2~32.9	73.8
19/09/2025	24.3~34.2	79.8
20/09/2025	21.5~26.7	78.4
21/09/2025	20.6~24.7	77.4
22/09/2025	21.7~31.8	62.5
23/09/2025	24.1~31.4	71.7
24/09/2025	24.8~30.5	87.3
25/09/2025	24.1~33.5	76.0
26/09/2025	23.3~34.3	72.5
27/09/2025	24.7~34.9	70.6
28/09/2025	26.5~35.5	68.1
29/09/2025	24.7~34.7	69.7
30/09/2025	25.1~34.8	76.9
01/10/2025	23.5~32.4	74.5
02/10/2025	25.4~36.5	64.1
03/10/2025	27.0~34.9	66.2
04/10/2025	24.5~35.6	63.9
05/10/2025	26.0~37.6	62.9
06/10/2025	28.0~38.3	60.5
07/10/2025	29.1~37.7	63.1

08/10/2025	27.3~35.1	60.7
09/10/2025	24.5~34.9	55.0
10/10/2025	22.6~36.6	53.5
11/10/2025	24.5~37.4	56.9
12/10/2025	24.7~37.3	57.8
13/10/2025	27.3~36.1	64.1
14/10/2025	23.4~26.7	78.1
15/10/2025	22.7~30.9	69.6
16/10/2025	24.3~29.4	75.5
17/10/2025	23.9~32.6	71.9
18/10/2025	20.2~26.7	80.6
19/10/2025	16.4~20.0	68.6
20/10/2025	15.1~17.5	61.7
21/10/2025	13.6~15.5	65.2
22/10/2025	13.0~18.4	58.3
23/10/2025	13.0~20.3	53.5
24/10/2025	12.4~21.3	58.6
25/10/2025	13.0~23.2	60.1
26/10/2025	14.2~21.4	60.4
27/10/2025	14.9~21.4	55.0
28/10/2025	13.3~22.2	61.0
29/10/2025	11.8~25.3	58.0
30/10/2025	17.1~19.6	76.7
